# Short stump and high anastomosis pull-through (SHiP) procedure for delayed coloanal anastomosis with no protective stoma for low rectal cancer

**DOI:** 10.1007/s13304-021-01022-6

**Published:** 2021-03-16

**Authors:** Francesco Bianco, Paola Incollingo, Armando Falato, Silvia De Franciscis, Andrea Belli, Fabio Carbone, Gaetano Gallo, Mario Fusco, Giovanni Maria Romano

**Affiliations:** 1 General Surgery Unit, San Leonardo Hospital, Castellammare di Stabia, ASL NA3 Sud, Viale Europa, 283, 80053 Naples, Italy; 2 General Surgery Unit, Department of Advanced Biomedical Sciences, University Federico II, San Leonardo Hospital, Castellammare di Stabia, ASL NA3 Sud, Naples, Italy; 3 General and Laparoscopic Surgery Unit, San Giuliano Hospital, Giugliano in Campania, Naples, Italy; 4 Istituto Nazionale Tumori IRCCS Fondazione G. Pascale, Naples, Italy; 5 Department of Advanced Biomedical Sciences, University Federico II, Naples, Italy; 6 Department of Medical and Surgical Sciences, University of Catanzaro, Catanzaro, Italy; 7 Cancer Registry Unit, ASL NA3 Sud, Torre del Greco, Italy; 8 San Michele Hospital, Maddaloni, Italy

**Keywords:** Pull- through, Coloanal anastomosis, Rectal cancer, Stoma, Turnbull–Cutait

## Abstract

Despite advances in coloanal anastomosis techniques, satisfactory procedures completed without complications remain lacking. We investigated the effectiveness of our recently developed ‘Short stump and High anastomosis Pull-through’ (SHiP) procedure for delayed coloanal anastomosis without a stoma. In this retrospective study, we analysed functional outcomes, morbidity, and mortality rates and local recurrence of 37 patients treated using SHiP procedure, out of the 282 patients affected by rectal cancer treated in our institution between 2012 and 2020. The inclusion criterion was that the rectal cancer be located within 4 cm from the anal margin. One patient died of local and pulmonary recurrence after 6 years, one developed lung and liver metastases after 2 years, and one experienced local recurrence 2.5 years after surgery. No major leak, retraction, or ischaemia of the colonic stump occurred; the perioperative mortality rate was zero. Five patients (13.51%) had early complications. Stenosis of the anastomosis, which occurred in nine patients (24.3%), was the only long-term complication; only three (8.1%) were symptomatic and were treated with endoscopic dilation. The mean Wexner scores at 24 and 36 months were 8.3 and 8.1 points, respectively. At the 36-month check-up, six patients (24%) had major LARS, ten (40%) had minor LARS, and nine (36%) had no LARS. The functional results in terms of LARS were similar to those previously reported after immediate coloanal anastomosis with protective stoma. The SHiP procedure resulted in a drastic reduction in major complications, and none of the patients had a stoma.

## Introduction

Coloanal anastomosis remains the subject of lively clinical debate despite modern advances in the technique, given that outcomes remain unsatisfactory [1–4]. Since Rullier et al. published their criteria in 2013, there has been a notable increase in the rate of surgeries that conserve the pelvic floor, thereby promoting the possibility of expanding this type of reconstruction to all patients who have no invasion of the pelvic floor muscles [5]. Nevertheless, none of the coloanal anastomosis procedures used to date is without notable complications; they usually do not provide satisfactory functional outcomes [6, 7].

In this study, we investigated the effectiveness of our recently developed ‘Short stump and High anastomosis Pull-through’ (SHiP) procedure for delayed coloanal anastomosis without a stoma. This method represents a modification of the previously described pull-through procedure [8–11] that attempts to address the aforementioned limitations. The development of this technique dates back to 2012 as reported in previous studies [12–14]; we refined this procedure based on our experience with 37 consecutive patients via continuous modifications.

In this retrospective study, we analysed the functional outcomes as well as the morbidity and mortality rates of 37 patients treated for low rectal cancer using the SHiP procedure out of the 282 patients affected by rectal cancer treated in our institution between 2012 and 2020.

## Materials and methods

This was a retrospective single-centre study and is reported according to the Strengthening the Reporting of Observational Studies in Epidemiology (STROBE) statement for cohort studies [15].

The ethics committee of our institution approved this study. Between 2012 and 2020, 37 patients were treated for low rectal cancer using the SHiP procedure out of the 282 patients affected by rectal cancer.

The inclusion criterion were as follows: rectal cancer be located within 4 cm from the anal margin, no radiological sign of cancer invasion of the internal sphincter and/or levator ani muscle, strong motivation of the patient to avoid temporary ileostomy. All patients were informed of alternative surgical treatments and provided written informed consent regarding the procedure and treatment of personal data.

**Fig. 1:**
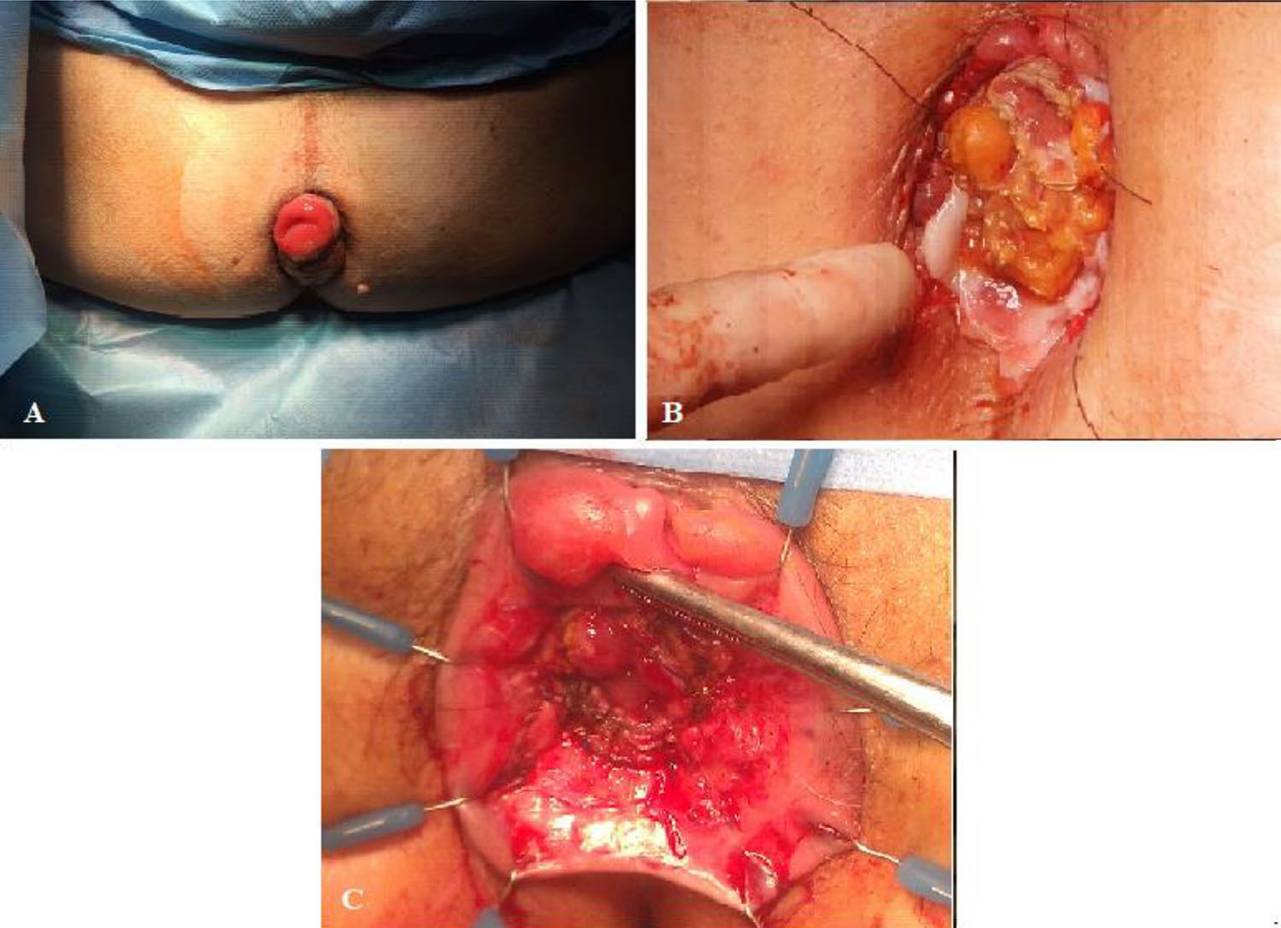
Second stage: resection of the colic stump and fashioning of the high coloanal anastomosis; **a** stump at the end of the first stage; **b** identification of the referral stitches, adhesiolysis, section of the stump and completion of the High coloanal anastomosis; **c** high coloanal anastomosis at the end of the second stage of the procedure

The surgical technique carried out is a conventional low anterior resection, including high vascular ligation, complete left colon, splenic flexure and half transverse colon mobilization (up to middle colic vessels) and total mesorectal excision. Anal mucosectomy and transanal rectal section are then performed and the left colonic stump is pulled through the anus. Four referral stitches (which will be the markers for fashioning the delayed anastomosis) are placed between the colic serosa and the upper verge of the anal canal (cranially to the internal sphincter). In the second stage of the procedure the adhesions between the colonic stump and the anal canal are bluntly dissected until the marker stitches. The colonic stump is then sectioned at this level, leaving the anal canal free from the residual colon, and a “high” anastomosis completed with four additional stitches [12, 14]. In order to minimize the discomfort between the two steps of the procedure, in the second half of the study, the length of the stump was reduced and a short stump of approximately 2 cm-length from the anocutaneous line was performed (Fig. 1). Thirty-seven patients (25 men and 12 women) aged between 38 and 74 years underwent the procedure; preoperative patient data (American Society of Anesthesiologists score, body mass index, and comorbidities) are shown in Table 1. Any cardiovascular and respiratory disease, obesity, kidney failure, diabetes and cerebral ischaemia were considered as comorbidities. Two patients had undergone previous recto-anal surgeries for benign disease (hemorrhoidectomy and transanal fistula excision), one had undergone a right hemicolectomy for cancer, and two previously underwent local transanal adenocarcinoma excision that required radical surgery. Thirty-two patients (86.5%) were administered neoadjuvant therapy before surgery (30 and two with long- and short-course chemoradiotherapy, respectively). All patients were instructed not to sleep in the supine position or to sit without dedicated supports during the period between the two surgical steps to avoid damage to the colonic stump. Patients returned for follow-up visits 1, 3, 6, 12, 18, and 24 months after surgery and every year thereafter.

**Table 1:** Patients’ anagraphic data

Age at diagnosis (mean)	60.8 (±8.82)		
BMI (mean)	25.7 (±3.74)		
Sex (m/f)	25/12		
Preop CRT (y/n)	32/5		
ASA (no.)	I	II	III
	2	23	12
Comorbidity (no.)	None (0)	Low (1)	High (2 or more)
	22	8	7

At each follow-up, patients underwent rectal digital exploration and the Cleveland Clinic Incontinence Score (Wexner score) and low anterior resection syndrome (LARS) score were used to evaluate Anal continence and function [16–19].

Two patients had an average follow-up of less than 1 year and were, therefore, not included in the statistical analyses of the Wexner and LARS scores.

## Results

The mean age of the patients was 60.8 years (range 38–74). The mean BMI was 25.7 ± 3.74 (range 18–38). Perioperative data are summarized in Table 2. Histological results after resection are reported in Table 3. Considering patient’s comorbidities, BMI, ASA score, previous abdominal surgery, tumor size, and hostile anatomy, laparotomy was performed in 23 (62%) patients while 11 (30%) underwent a laparoscopic approach and 3 (8%) a robotic approach (Table 2). The second surgical step (resection of the transanal colonic stump) was performed an average of 13.8 ± 4.3 days later; 22 patients were discharged between the 5th and 7th postoperative days and were then readmitted for 2 days to undergo the second step. As such, the mean total length of hospital stay was 8.2 days. Some of the first 18 patients experienced anastomotic stenosis; therefore, anal dilatators were routinely used in the first 6 months after surgery for all successive patients (Table 3). Furthermore, since no stump retraction was observed in the first half of the study (i.e., the first 18 patients), a short stump of approximately 2 cm length from the anocutaneous line was performed in all subsequent patients to reduce discomfort between the two steps of surgery (Fig. 1).

**Table 2:** Surgical data

Surgical tech (no.)	Open	Laparoscopic	Robotic
	23	11	3
Distance from anal verge (no.)	4 cm	3 cm	2 cm
	23	9	5
Interval between the two surgical steps (days mean)	13.8 (±4.28)		
Ileostomy (no.)	0

**Table 3:** Postoperative data and complications

Procedure failure	None
Perioperative mortality	None
Stump retraction/ischaemia	None
Coloanal leak	None
30 days compl sec. Dindo (no.)	
I	1
II	2
III A	1
III B	1
Long-term complications	Stenosis = 9
Stage (AJCC vs.7) (no.)	
Stage 0	6
Stage I	27
Stage II A	2
Stage III A	1
Stage III B	1

One patient died of local and pulmonary recurrence after 6 years, another developed lung and liver metastases after 2 years, and a third experienced local recurrence 2.5 years after surgery. None of the patients experienced failure of the surgical technique (Table 3). There were no incidences of major leaks, retractions, or ischaemia in the colonic stump; moreover, no perioperative fatalities occurred. Five patients (13.51%) had early complications within 30 days of surgery: three patients (one and two with Clavien–Dindo grades of I and II, respectively) were treated conservatively with medical therapy, another underwent relaparotomy for median suture dehiscence (Clavien–Dindo IIIB), and the fifth underwent computed tomography-guided percutaneous drainage of a pelvic abscess (Clavien–Dindo IIIA) [20]. Stenosis of the anastomosis was the only long-term complication observed. Stenosis on physical examination or colonoscopy was found in nine patients (24.3%); of these, only three (8.1%) were symptomatic and were treated with endoscopic dilatations, while the remaining six were asymptomatic and were treated with anal dilators. Following the routine introduction of anal dilators, only three patients developed anastomotic stenosis owing to their non-compliance regarding the use of prescribed dilators. The postoperative complications are listed in Table 3. Two patients were prescribed anti-diarrheal drugs 6 months after surgery.

**Fig. 2:**
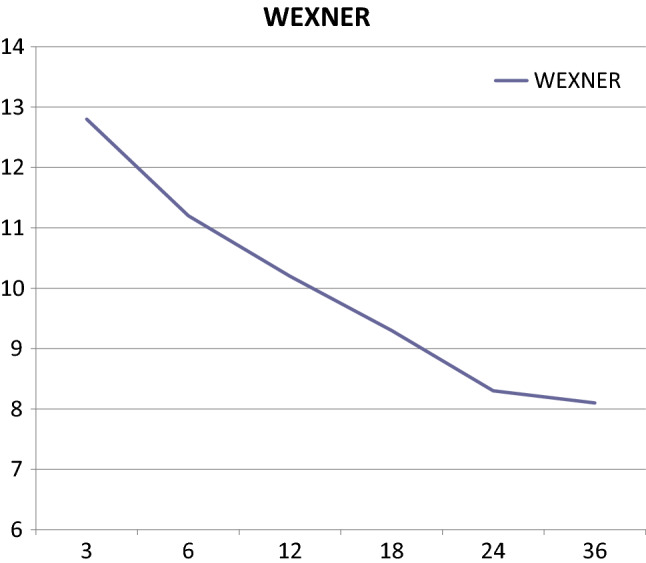
Wexner score trend over time

**Fig. 3:**
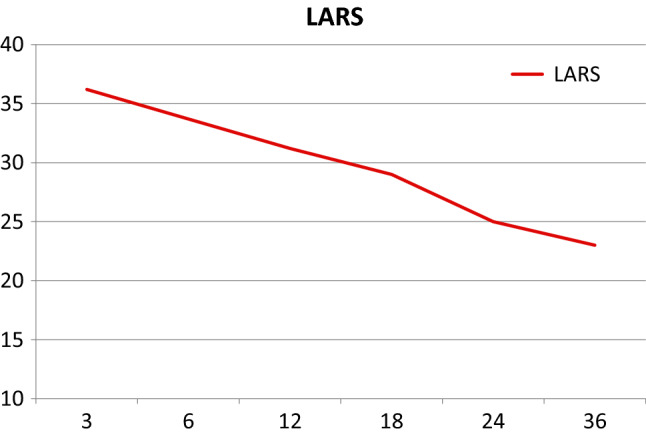
Low anterior resection syndrome score trend over time

The mean Wexner scores at 12, 24, and 36 months were 10.2, 8.3, and 8.1, respectively. The 57 and 30% of the patients had major LARS at 12 and 24 months, respectively. At the 36-month check-up, six patients (24%) had a major LARS, 10 (40%) had minor LARS, and nine (36%) had no LARS. The scores are fully reported in Table 4 (Figs. 2, 3).

**Table 4:** Functional results expressed by the means of the Wexner score and LARS score (Low Anterior Resection Syndrome score)

	12 M	24 M	36 M
WEXNER (mean)	10.2 (± 3.9)	8.3 (± 4.7)	8.1 (± 4.8)
LARS (mean)	31.1 (± 4.9)	24.8 (± 8.2)	23.2 (± 9.3)
MAJOR LARS (no.)	20 (57%)	9 (30%)	6 (24%)
MINOR LARS (no.)	15 (43%)	15 (50%)	10 (40%)
NO LARS (no.)	0 (0%)	6 (20%)	9 (36%)

No LARS = from 0 to 20, minor LARS = from 21 to 29, major LARS = from 30 to 42

## Discussion

Rullier et al. indicated that it is possible to reconstruct pelvic floor muscles with full anatomic integrity if they are free from neoplasia via sphincter-saving coloanal anastomosis [5]. Thus, it is possible to achieve such reconstruction while abolishing the need for a few centimeters’ of free margin; however, the ideal ultra-low coloanal anastomosis method (hand-made or stapled) remains the subject of debate [1–3].

To date, a high proportion of patients experience postoperative complications due to anastomotic leaks of coloanal anastomoses or to secondary events following reversal surgery for the closure of protective ileostomies [21–25]. Therefore, it is necessary to identify anastomosis techniques that can greatly reduce the rate of postoperative complications, which currently occur in up to 40% of patients with coloanal anastomoses and can delay or even prevent patients’ access to adjuvant chemotherapy and risk forming a definitive stoma [22, 26, 27]. As a consequence, patients with ultra-low coloanal anastomoses still require a temporary derivative stoma [28] which significantly affects their quality of life (particularly during chemoradiotherapy) [29].

Owing to such considerations, the impetus for this study was the need for a technique that would create the anastomosis only after the transposed colon had attached to the plane of the pelvic muscles, without risking leakage. These considerations were also the bases for the development of the pull-through technique [8, 9], which was abandoned in the 1980s following the introduction of mechanical staplers.

In the previously used pull-through technique, the colonic stump was resected after a period varying from 7 to 14 days, giving time to the serosa to adhere to the anal canal muscles (delayed coloanal anastomosis) and prohibiting the detachment of these adhesions in the second surgical step [11]. The latter is the main technical distinction from the modified technique that we have now adopted. In the SHiP procedure the adhesions are partially detached up to the landmark stitches allowing a high proximal stump resection and anastomosis, as described in our previous studies [12, 14].

The old pull-though procedure had some drawbacks that led to its gradual abandonment. The first limitation was sphincter incontinence due to the presence of a residual colonic stump inside the anal canal, which impeded the correct contraction of the muscles. That was a consequence of the technical prohibition against detaching the adhesions, which frequently led in the old procedure to a very low resection of stump very close to anocutaneous margin. In both the old procedure as well as the technique more recently described by French authors in 2011, it was forbidden to sever the adhesions inside the anal canal to avoid the risk of entering the pelvic cavity by detaching the colonic stump. This has led to the presence of a colonic remnant in the anal canal that affected sphincter contraction and continence [11, 30].

The second drawback of the old procedure was the risk of anastomotic leak [10, 31–33] and of ischaemia in the colonic stump transposed in the anal canal, the so-called “guillotine effect”. This was owing to the discrepancy between the tightening of the anal canal muscles and the mesocolonic diameters, as well as to terminal vascularization at the sigmoid level [12, 31].

The third drawback was the occurrence of stenosis in the coloanal anastomosis after the second step of the colonic stump resection [31].

Finally, the fourth drawback was patient discomfort during the 7- to 14-day period between the first and second steps of the procedure caused by the encumbrance of the colonic stump itself.

**Fig. 4:**
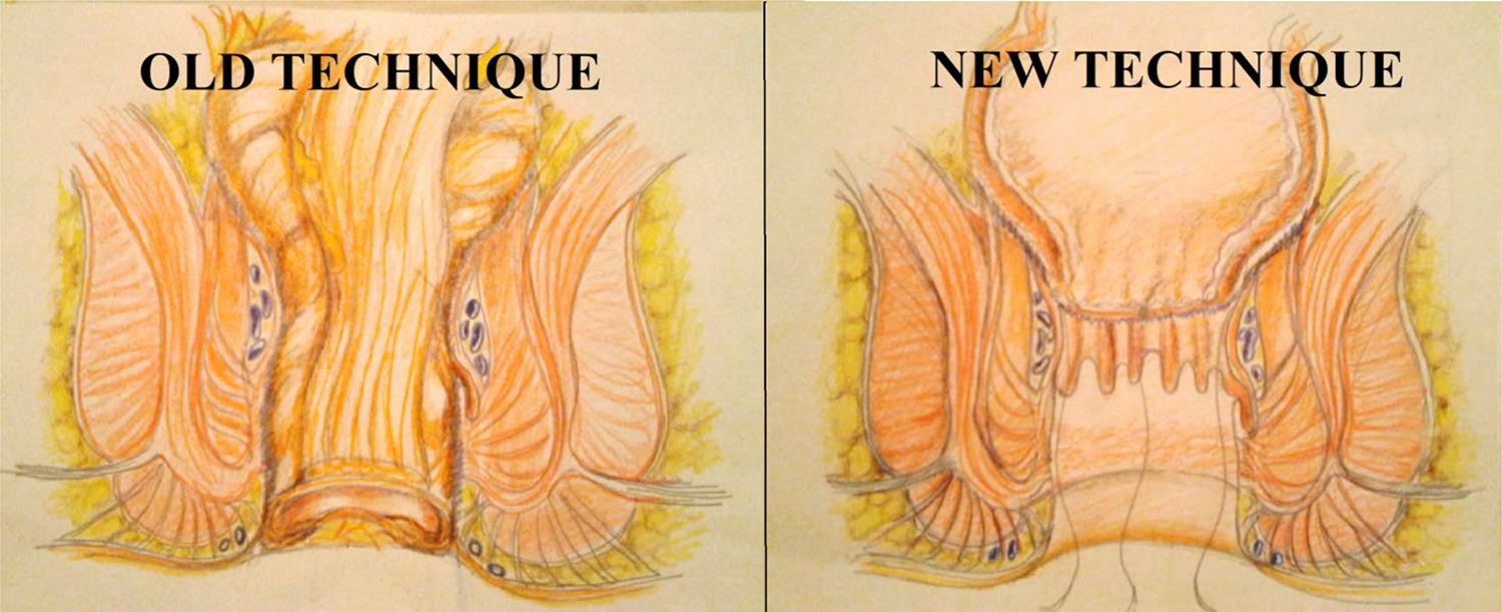
Schematic representation of the differences between the old and the new pull-through technique

Our experience with 37 consecutive patients permitted, through “continuous modifications”, the development of our modified SHiP procedure for delayed coloanal anastomosis without a protective stoma; this overcame all the drawbacks of the old procedure (Fig. 4).

As described in our previous papers [12–14] the technical modifications that were hallmarks of the new procedure included the placement of very proximal suture “markers” in the anal canal during the first step of the procedure. This allowed creating a coloanal anastomosis that was very high and proximal to the anal canal in the second step, thereby freeing the anal canal from the residual colonic stump and allowing better sphincter activity. Additionally, the complete mobilization of the left colon up to the middle colonic vessels allowed for transposition of the left colon in the anal canal, whose vascularization (in contrast to that of the sigmoid colon) is arched and not terminal. This latter and the minor diameter discrepancy with the anal canal of the left mesocolon as compared to the meso-sigmoid prevented ischaemia of the colonic stump. This extended mobilization also allows the left colon to lie floppily and tension-free in the pelvis along the sacral curve, thus avoiding any retraction of colonic stump [12, 14].

These modifications made it possible to avoid the risk both of anastomotic leaks and colonic stump ischaemia, thereby making a protective stoma unnecessary. The favourable results obtained after our initial experiences made it possible to realize that it was not necessary to create an overly long colonic stump as required by the old technique; instead, a stump of a few centimeters (similar to the size of a mucosal rectal prolapse) is sufficient (Fig. 1).

Shortening the colonic stump prevents considerable discomfort caused by the stump itself between the two steps of the procedure. The operations were performed as open, laparoscopic, and robotic surgeries; hence, it was possible to avoid scarring with no abdominal incision while the specimen was extracted through the anus. Patients with bulky tumors were excluded from the non-scarring procedure, as extraction through the anus would cause excessive stretching and damage of the sphincter; in such cases, the specimens were extracted through an abdominal incision.

None of the 37 patients who underwent surgery with the modified technique experienced postoperative mortality or anastomotic leaks, and only one patient underwent re-laparotomy for median suture dehiscence. Moreover, only two patients experienced local recurrence, and one died of metastases.

Stenosis of the anastomosis occurred in nine patients, among whom three were treated with endoscopic dilation and the remaining six utilized anal dilators as outpatients. This experience prompted us to use anal dilators starting from 30 days up to the 6th month after surgery (until the anastomosis was stable); this made it possible to eliminate stenosis in all patients and to improve functional results.

As previously reported, foecal incontinence and LARS progressively improve during the 2 years after surgery in patients with immediate coloanal anastomosis; there are no differences in functional outcomes between patients who undergo straight (end-to-end) or lateral (side-to-end) anastomosis and those who undergo reconstruction via the J-pouch procedure after 1 year has passed [34]. Even in our experience, patients undergoing the SHiP procedure had progressive improvement in continence and LARS up to 3 years post-surgery (Table 4; Figs. 2, 3).

The functional results obtained using the SHiP as measured using the LARS and Wexner scores were at least similar to those reported in the literature after immediate coloanal anastomosis, but without the need for any protective ileostomy [30, 34–37]. Compared to immediate coloanal anastomosis, the SHiP procedure allowed for a drastic reduction in major complications despite the fact that none of the patients had a definitive stoma. Worse functional results were achieved in patients with high body mass indices, in the one who had a previous right hemicolectomy, and women with more natural childbirths. This suggested a need for more careful selection of patients.

Previously published data from patients who underwent immediate coloanal anastomosis with a protective stoma reveal that 20–40% of them had major complications with temporary protective stomas becoming definitive [22–24] as well as a proportion that developed metabolic complications [38]. Furthermore, favourable functional outcome rates were inversely proportional to postoperative complication rates related to the coloanal anastomosis. As such, the marked reduction in complications when using the SHiP procedure reflects in an improvement in long-term functional results.

The greatest improvement is undoubtedly in terms of patient satisfaction, as subjects will not require a stoma (even for a limited period of time) nor will they require another intra-abdominal operation for stoma reversal.

The aspects described above have an important impact on deciding whether to perform SHiP or an immediate coloanal anastomosis with protective ileostomy. It is important to highlight that some patients will undergo postoperative adjuvant chemotherapy; therefore, the occurrence of complications due to the first and/or reversal surgery might force the patient to undergo chemotherapy with a stoma, postponing its closure and thus markedly worsening the patient’s quality of life [39, 40].

Our study has some limitations. It is a single-centre study with lack of a control group. Although the data were collected prospectively, data analysis was performed retrospectively. Further randomized trials will be needed [41].

## Conclusion

The functional outcomes obtained using the SHiP procedure were at least on par with those reported in the literature after immediate coloanal anastomosis in terms of LARS. Indeed, the SHiP procedure resulted in a drastic reduction in major complications, and none of the patients required a stoma. Results from multicenter randomized controlled trials are now warranted. Nevertheless, our experience over 8 years of practice, and the observed outcomes both in terms of functional results and complications, indicate that the SHiP procedure might play a fundamental role in the treatment of ultra-low rectal cancer.

## Data Availability

All data generated or analysed during this study are included in this published article.
